# Subfascial infiltration of 0.5% ropivacaine, but not 0.25% ropivacaine, exacerbates damage and inflammation in surgically incised abdominal muscles of rats

**DOI:** 10.1038/s41598-022-13628-w

**Published:** 2022-06-07

**Authors:** Dandan Shen, Yuki Sugiyama, Kumiko Ishida, Satoshi Fuseya, Takashi Ishida, Mikito Kawamata, Satoshi Tanaka

**Affiliations:** Department of Anesthesiology and Resuscitology, Shinshu University School of Medicine, Matsumoto City, Nagano 390-8621 Japan

**Keywords:** Drug safety, Pain, Pain management

## Abstract

Ropivacaine-induced myotoxicity in surgically incised muscles has not been fully investigated. We evaluated the effects of infiltration anesthesia with ropivacaine on damage, inflammation and regeneration in the incised muscles of rats undergoing laparotomy. Ropivacaine or saline was infiltrated below the muscle fascia over the incised muscles. Pain-related behaviors and histological muscle damage were assessed. Macrophage infiltration at days 2 and 5 and proliferation of satellite cells at day 5 were detected by CD68 and MyoD immunostaining, respectively. Pain-related behaviors were inhibited by 0.25% and 0.5% of ropivacaine for 2 h after surgery. Single infiltration of 0.5% ropivacaine did not induce injury in intact muscles without incision, but single and repeated infiltration of 0.5% ropivacaine significantly augmented laparotomy-induced muscle injury and increased the numbers of CD68-positve macrophages and MyoD-positive cells compared to those in rats with infiltration of saline or 0.25% ropivacaine. In contrast, there were no significant differences in them between rats with saline infusion and rats with 0.25% ropivacaine infiltration. In conclusion, single or repeated subfascial infiltration of 0.25% ropivacaine can be used without exacerbating the damage and inflammation in surgically incised muscles, but the use of 0.5% ropivacaine may be a concern because of potentially increased muscle damage.

## Introduction

Surgical site infiltration of local anesthetics has been shown to improve postoperative analgesia and reduce opioid requirements in various types of surgery including not only minimally invasive surgery such as laparoscopic surgery but also more extensive surgical procedures such as open abdominal surgery^1–3^. Wound Infiltration analgesia at the surgical site is easy to perform, and this technique has therefore become popular in clinical practice. In the abdominal wall consisting of skin, fascia, muscles and peritoneum, subfascial delivery of local anesthetics has been shown to be more effective than subcutaneous delivery for analgesia^4,5^.

It is well known that clinically relevant concentrations of local anesthetics can induce myotoxicity. For example, direct injection of 0.5% bupivacaine, 0.5% levobupivacaine or 0.5% ropivacaine into intact skeletal muscles has been shown to have myotoxic and apoptotic effects on the muscles in animals^6,7^. However, the myotoxic effects of local anesthetics at lower concentrations on incised muscles have not been investigated yet.

Relatively large amounts of local anesthetics are used for local infiltration anesthesia to block the nerve branches innervating the extent of surgically injured tissues^2^. Thus, not only intact muscles but also surgically incised muscles are exposed to local anesthetics. Most types of skeletal muscle injury follow a healing process consisting of three distinct phases: acute inflammatory and degenerative phase, regeneration phase, and maturation and remodeling phase^8^. It is clinically essential to investigate the safety of local anesthetics for skeletal muscles in various states. We hypothesized that local anesthetics-induced myotoxicity might vary depending on their concentrations and whether the muscle tissue is in an intact or injured condition.

The purpose of the present study was thus to investigate, by using a rat laparotomy model, the effects of subfascial infiltration of ropivacaine, a local anesthetic commonly used in clinical practice, at concentrations of 0.25% and 0.5% on damage, inflammation, and regeneration of surgically incised muscle tissues. We also examined the analgesic effects of ropivacaine infiltration on post-laparotomy pain in rats.

## Results

### Experiment 1: Analgesic effects of single subfascial infiltration of ropivacaine after laparotomy

**Figure 1:**
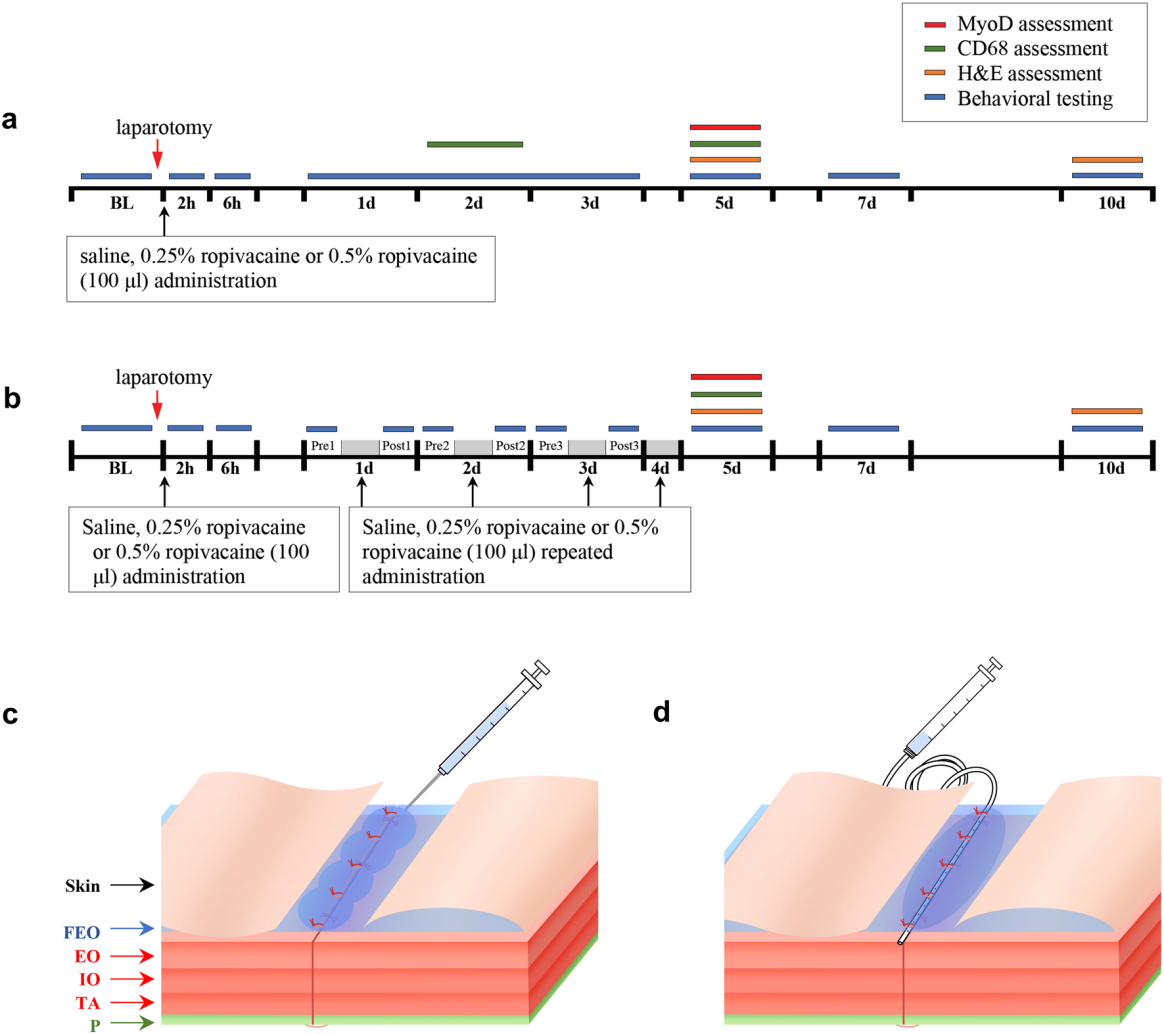
Experimental design and the administration site of the allocated drug. (**a**) Protocol of experiment 1(single dose administration) and (**b**) protocol of experiment 2 (repeated dose administration). Pain-related behaviors were observed to assess analgesic effects of the drugs. H&E staining was performed to evaluate muscle damage. An anti-CD68 antibody was used to assess macrophage infiltration, and an anti-MyoD antibody was used to detect proliferation of satellite cells. (**c**) In experiment 1, abdominal wall muscles, including EO, IO and TA, and P were reapproximated and FEO was sutured. Subfascial administration of 100 μl of 0.25% ropivacaine, 0.5% ropivacaine or saline was done along the incisional line of the muscles. (**d**) In experiment 2, a 15-cm-long multiorifice catheter with 15 side-holes evenly within 2 cm from the top was subfascially placed along the incisional line of the muscle to administer ropivacaine or saline repeatedly. Then the muscle fascia was closed and sutured to the catheter. BL indicates baseline; H&E, hematoxylin and eosin; CD68, a marker of macrophage infiltration; MyoD, a marker of satellite cell proliferation; FEO, fascia of the external oblique muscle; EO, external oblique muscle; IO, internal oblique muscle; TA, transversus abdominis muscle; P, parietal peritoneum.

**Figure 2:**
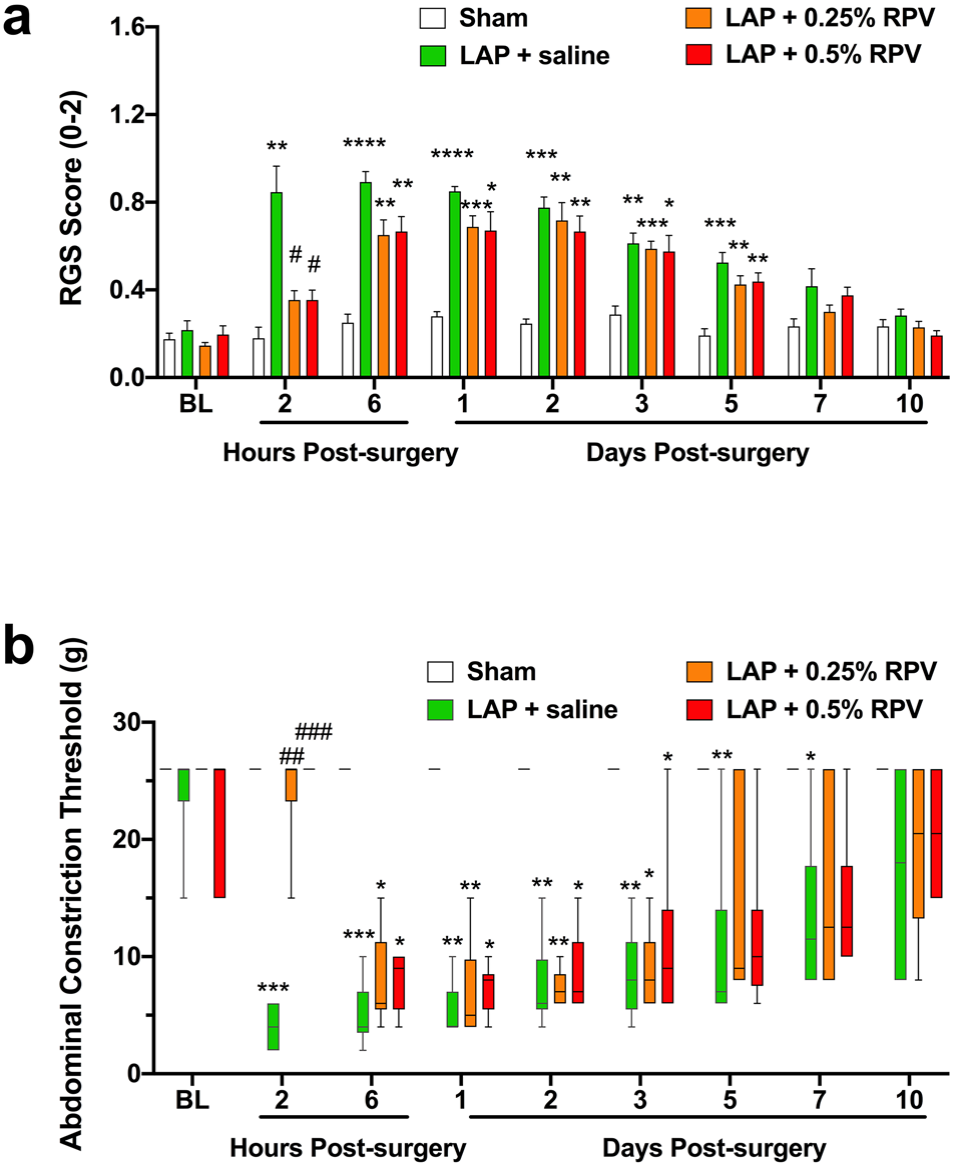
Effects of single subfascial infiltration of saline, 0.25% ropivacaine and 0.5% ropivacaine on RGS and ACT after laparotomy. (**a**) Spontaneous pain assessed by RGS score and (**b**) changes in ACTs stimulated by von Frey filaments were measured before laparotomy and 2 h, 6 h, 1d, 2d, 3d, 5d, 7d and 10d after laparotomy. Data for RGS scores were analyzed by two-way ANOVA with Tukey’s post hoc comparisons, and are presented as means ± SEM. Data for ACTs were analyzed by the Kruskal–Wallis test with Dunnett’s post hoc comparisons, and are presented as the median with first and third quartiles. n = 6 in each group. RGS scores and ACTs were suppressed for 2 h after single infiltrations of 0.25% and 0.5% ropivacaine in the surgically incised muscles. *P < 0.05, **P < 0.01, ***P < 0.001, ****P < 0.0001 vs sham group at each time point, #*P* < 0.05, ##*P* < 0.01, ###*P* < 0.001 vs LAP + saline group at each time point. See Supplementary Table S1 for details on statistics. RPV indicates ropivacaine; LAP, laparotomy; BL, baseline; RGS, rat grimace scale; ACT, abdominal constriction threshold; SEM, standard error of the mean; ANOVA, analysis of variance.

Analgesic effects of single subfascial infiltration of saline, 0.25% and 0.5% ropivacaine on pain-related behaviors including the scores of Rat Grimace Scale (RGS) and Abdominal constriction threshold (ACT) in rats were evaluated (Fig. 1a,c). RGS in the laparotomy + 0.25% ropivacaine (LAP + 0.25% RPV) group and laparotomy + 0.5% ropivacaine (LAP + 0.5% RPV) group were significantly lower than the score in the laparotomy + saline (LAP + saline) group only at 2 h post-surgery (*P* = 0.0284 and *P* = 0.0282, respectively) (Fig. 2a). ACT in the LAP + 0.25% RPV group and LAP + 0.5% RPV group only at 2 h after surgery were significantly higher than that in the LAP + saline group (*P* = 0.0047 and P = 0.0008, respectively) (Fig. 2b). There was no significant difference in RGS or ACT among the three groups thereafter. There was no significant difference in RGS or ACT between the LAP + 0.25% RPV group and LAP + 0.5% RPV group at any time point.

### Experiment 1: Hematoxylin and eosin (H&E) staining assay in and around the surgically incised muscle after single subfascial infiltration of ropivacaine.

**Figure 3:**
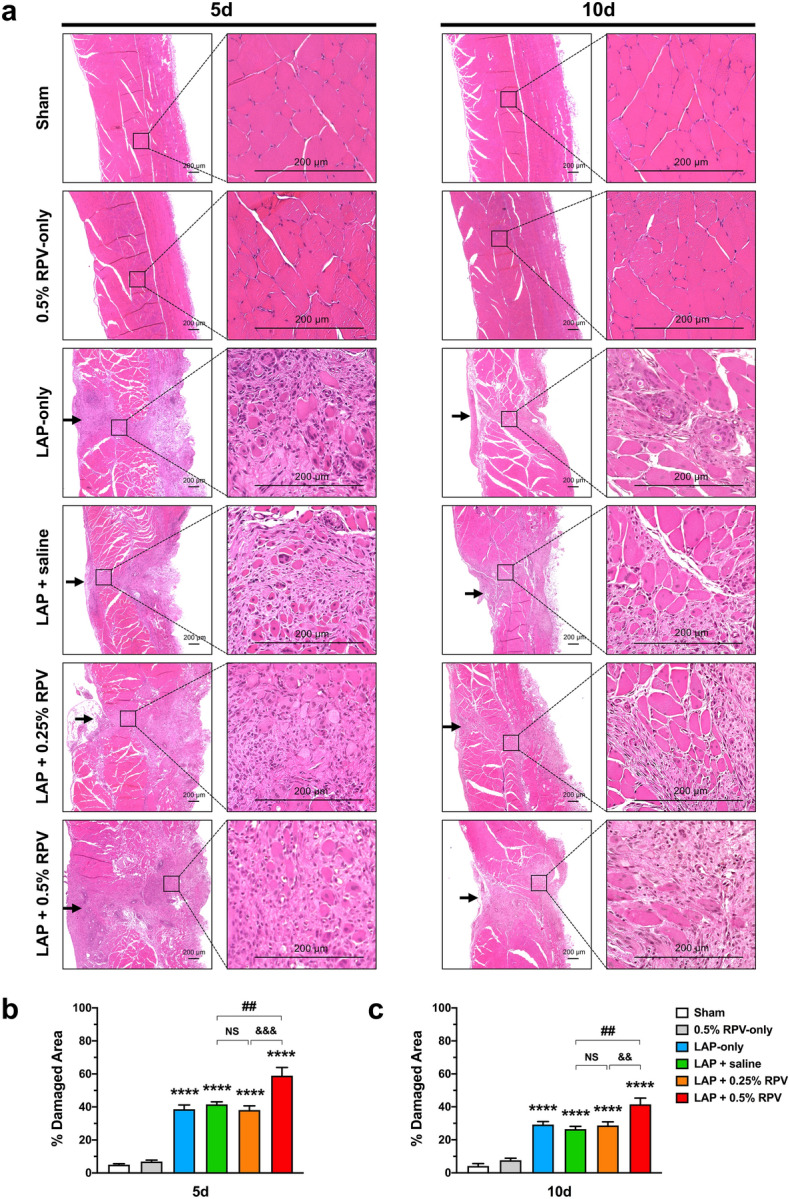
Damaged area in and around the incision site after single subfascial infiltration of saline or 0.25% ropivacaine or 0.5% ropivacaine. (**a**) Representative images of H&E-stained cross sections of the abdominal muscle tissues in and around the incision site at low magnification (left) and at high magnification (right) on days 5 and 10 after laparotomy. Areas of muscle loss after surgery except for sham surgery (sham) and 0.5% ropivacaine administrated below the fascia of intact muscles (0.5% RPV-only) were observed along the incision line. Black arrowheads show the incision line. Scale bar, 200 μm. Percents of damaged area in and around the incision site per area on (**b**) day 5 and (**c**) day 10. Data are presented as means ± SEM (n = 4–5 for each group) and were analyzed by one-way ANOVA for repeated measures with Tukey’s post hoc test. ****P < 0.0001 vs sham group, ##*P* < 0.01 vs LAP + saline group. Significant differences between the LAP + 0.25% RPV group and LAP + 0.5% RPV group was presented as &&*P* < 0.01 and &&&*P* < 0.001. See Supplementary Table S1 for details on statistics. RPV indicates ropivacaine; LAP, laparotomy; NS, no significant; H&E, hematoxylin and eosin; SEM, standard error of the mean; ANOVA, analysis of variance.

Damage of muscle tissues located along the incision site was evident at 5 days post-surgery and the percent of damaged area was decreased at 10 days post-surgery in each experimental group except for the sham group and subfascial injection of 0.5% ropivacaine without abdominal muscles incision (0.5% RPV-only) group (Fig. 3a). The percent of damaged area in the 0.5% RPV-only group was not significantly different from that in the sham group at day 5 or day 10 post-surgery. There were no significant differences in the percent of damaged area between the laparotomy without intervention (LAP-only), LAP + saline and LAP + 0.25% RPV groups at day 5 or day 10 post-surgery (Fig. 3b,c). In contrast, the percent of damaged area in the LAP + 0.5% RPV group at day 5 and 10 were significantly larger than those in the LAP + saline group (day 5, 59% ± 5% vs 42% ± 2%, *P* = 0.0023; day 10, 42% ± 4% vs 26% ± 2%, *P* = 0.0012) and LAP + 0.25% RPV group (day 5, 59% ± 5% vs 38% ± 3%, *P* = 0.0003; day 10, 42% ± 4% vs 29% ± 2%, *P* = 0.0061) (Fig. 3b,c).

### Experiment 1: Infiltration of CD68-positive cells in and around the surgically incised muscles after single subfascial infiltration of ropivacaine.

**Figure 4:**
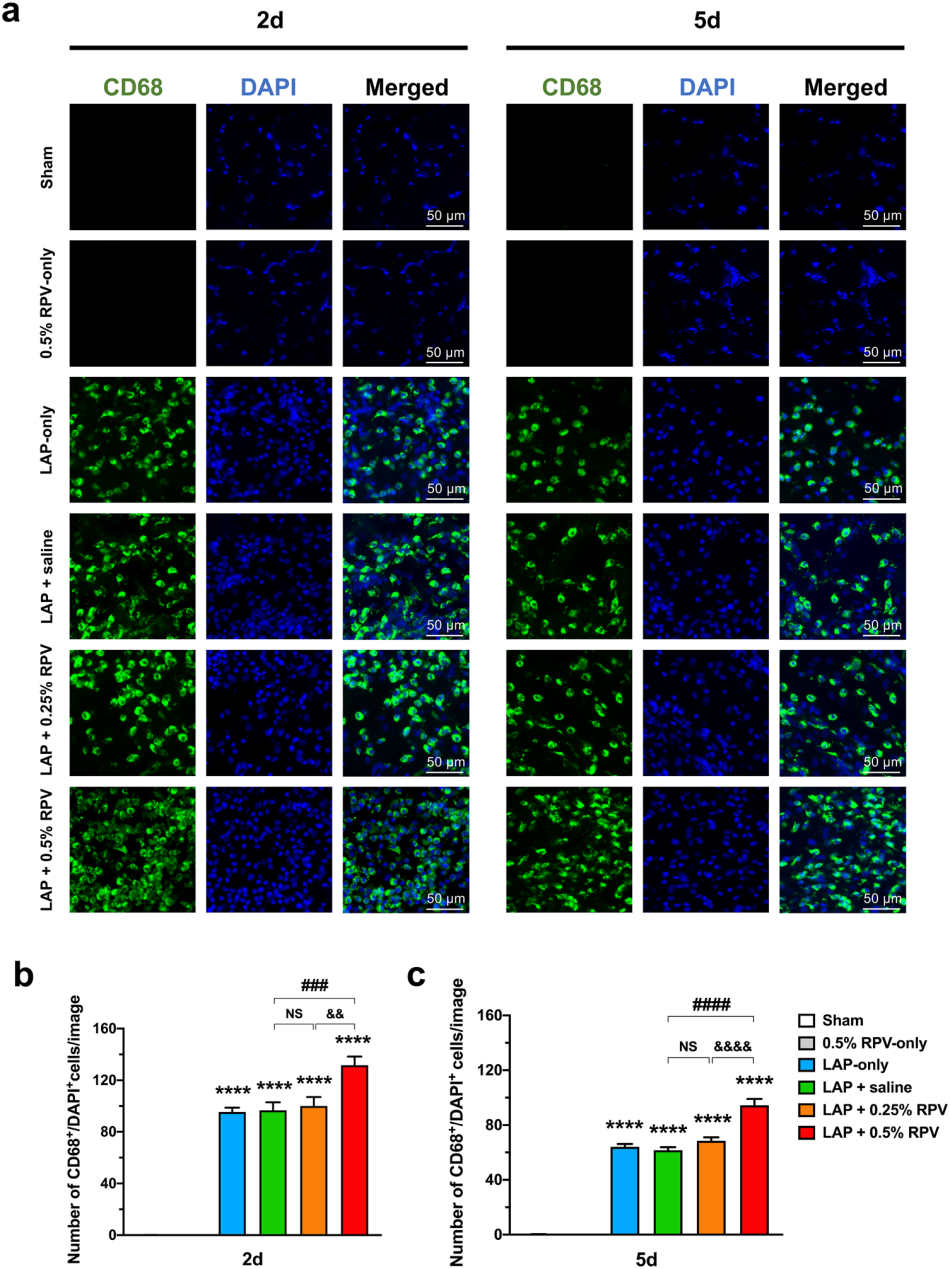
Infiltration of CD68-positive cells in and around the incision site after single subfascial infiltration of saline or 0.25% ropivacaine or 0.5% ropivacaine. (**a**) Representative images of immunofluorescence staining of CD68 in and around the incision site at high magnification at 2 and 5 days after laparotomy. Green, CD68; blue, DAPI. Scale bar, 50 μm. Numbers of CD68^+^/DAPI^+^ cells in and around the incision site were counted on (**b**) day 2 and (**c**) day 5 after laparotomy. Data are presented as means ± SEM (n = 4–5 for each group) and were analyzed by one-way ANOVA for repeated measures with Tukey’s post hoc test. ****P < 0.0001 vs sham group, ###*P* < 0.001, ####*P* < 0.0001 vs LAP + saline group. Significant differences between the LAP + 0.25% RPV group and LAP + 0.5% RPV group was presented as &&*P* < 0.01 and &&&&*P* < 0.0001. Typical examples of the distribution of CD68-positive cells in the entire field at low magnification were shown in Supplementary Figure S2. See Supplementary Table S1 for details on statistics. RPV indicates ropivacaine; LAP, laparotomy; NS, no significant; CD68, a marker of macrophage infiltration; DAPI, 4’,6-diamidino-2-phenylindole; SEM, standard error of the mean; ANOVA, analysis of variance.

There was no significant difference in the numbers of CD68^+^/DAPI^+^ cells between the sham group and 0.5% RPV-only group at day 2 or day 5 post-surgery. The numbers of CD68^+^/DAPI^+^ cells in the LAP + saline and LAP + 0.25% RPV groups were not significantly different from those in the LAP-only group at day 2 and day 5 post-surgery (Fig. 4a–c). In contrast, at day 2 post-surgery, the number of CD68^+^/DAPI^+^ cells in the LAP + 0.5% RPV group was significantly larger than that in the LAP + saline group (132 ± 7 vs 97 ± 6 per image, *P* = 0.001) and LAP + 0.25% RPV group (132 ± 7 vs 100 ± 7 per image, *P* = 0.0029) (Fig. 4b). There was also a significant difference between those two groups at day 5 post-surgery (LAP + 0.5% RPV group vs LAP + saline group, 94 ± 5 vs 62 ± 2 per image, *P* < 0.0001; LAP + 0.5% RPV group vs LAP + 0.25% RPV group, 94 ± 5 vs 69 ± 3 per image, *P* < 0.0001) (Fig. 4c).

### Experiment 1: MyoD expression in and around the surgically incised muscles after single subfascial infiltration of ropivacaine

**Figure 5:**
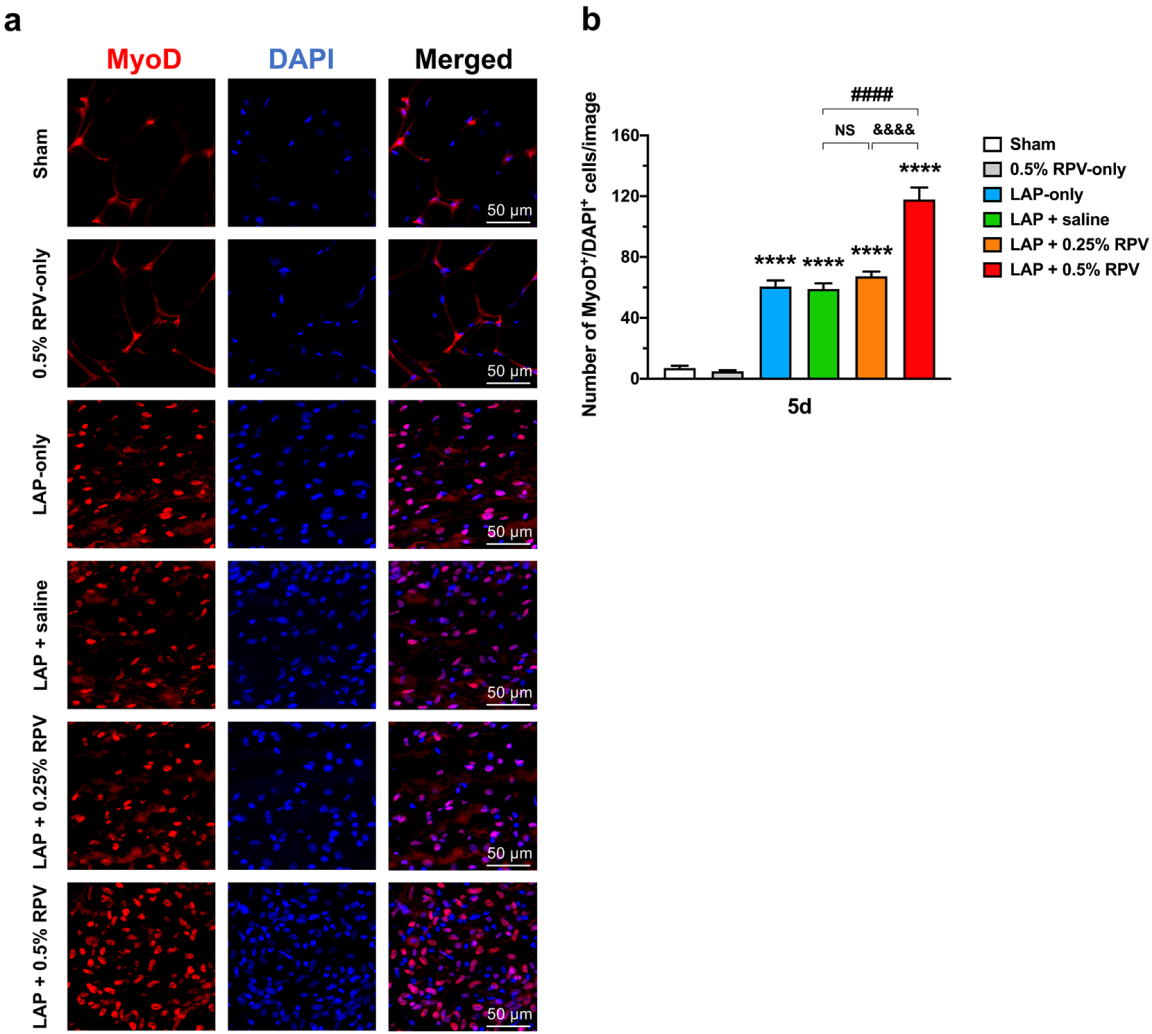
Expression of MyoD-positive cells in and around the incision site after single subfascial infiltration of saline or 0.25% ropivacaine or 0.5% ropivacaine. (**a**) Representative images of immunofluorescence staining of MyoD in and around the incision site at high magnification at 5 days after laparotomy. Red, MyoD; blue, DAPI. Scale bar, 50 μm. (**b**) Numbers of MyoD^+^/DAPI^+^ cells in and around the incision site were counted on day 5 after laparotomy. Data are presented as means ± SEM (n = 4–5 for each group) and were analyzed by one-way ANOVA for repeated measures with Tukey’s post hoc test. ****P < 0.0001 vs sham group, ####*P* < 0.0001 vs LAP + saline group. Significant difference between the LAP + 0.25% RPV group and LAP + 0.5% RPV group was presented as &&&&*P* < 0.0001. See Supplementary Table S1 for details on statistics. RPV indicates ropivacaine; LAP, laparotomy; NS, no significant; MyoD, a marker of satellite cell proliferation; DAPI, 4’,6-diamidino-2-phenylindole; SEM, standard error of the mean; ANOVA, analysis of variance.

The number of MyoD^+^/DAPI^+^ cells in the 0.5% RPV-only group was not significantly different from that in the sham group at day 5 post-surgery. No significant difference was found in the number of MyoD^+^/DAPI^+^ cells among the LAP-only, LAP + saline and LAP + 0.25% RPV groups. In contrast, the number of MyoD^+^/DAPI^+^ cells in the LAP + 0.5% RPV group was significantly larger than that in the LAP + saline group (118 ± 8 vs 59 ± 4 per image, *P* < 0.0001) and LAP + 0.25% RPV group (118 ± 8 vs 67 ± 3 per image, *P* < 0.0001) (Fig. 5a,b).

### Experiment 2: Analgesic effects of repeated subfascial infiltration of ropivacaine after laparotomy

**Figure 6:**
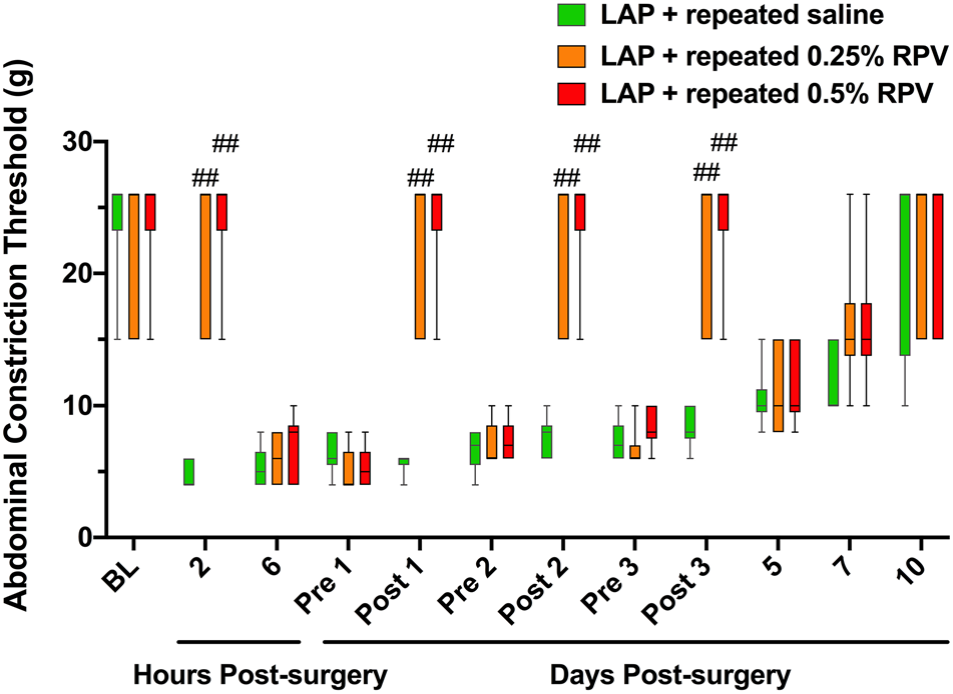
Effects of repeated subfascial infiltration of saline, 0.25% ropivacaine and 0.5% ropivacaine on ACT after laparotomy. Changes in ACTs stimulated by von Frey filaments were measured before laparotomy and 2 h, 6 h, pre 1d, post 1d, pre 2d, post 2d, pre 3d, post 3d, 5d, 7d and 10d after laparotomy. On days 1, 2 and 3 post-surgery, ACT was evaluated before (pre) and 2 h after (post) administration of saline, 0.25% ropivacaine or 0.5% ropivacaine. Data are presented as the median with first and third quartiles (n = 6 for each group) and were analyzed by the Kruskal–Wallis test with Dunnett’s post hoc comparisons. ACTs were inhibited repeatedly after repeated infiltrations of 0.25% ropivacaine or 0.5% ropivacaine in the surgically incised muscles. ##*P* < 0.01 vs LAP + repeated saline group at each time point. See Supplementary Table S1 for details on statistics. RPV indicates ropivacaine; LAP, laparotomy; BL, baseline; ACT, abdominal constriction threshold.

Analgesic effects of repeated subfascial infiltration of 0.25% and 0.5% ropivacaine on pain-related behaviors including ACT in rats were evaluated (Fig. 1b,d). Repeated administration of 0.25% ropivacaine and 0.5% ropivacaine significantly increased ACT after 2 h post-surgery compared with that in rats administrated saline (laparotomy + repeated administration of 0.25% ropivacaine (LAP + repeated 0.25% RPV): post 1d, *P* = 0.0056; post 2d, *P* = 0.0061; post 3d, *P* = 0.0061; laparotomy + repeated administration of 0.5% ropivacaine (LAP + repeated 0.5% RPV): post 1d, *P* = 0.0017; post 2d, *P* = 0.0019; post 3d, *P* = 0.0019) (Fig. 6). There was no significant difference in ACT between the LAP + repeated 0.25% RPV group and LAP + repeated 0.5% RPV group at any time point.

### Experiment 2: H&E staining assay, infiltration of CD68-positive cells and MyoD expression at the surgically incised muscles after repeated subfascial infiltration of ropivacaine.

**Figure 7:**
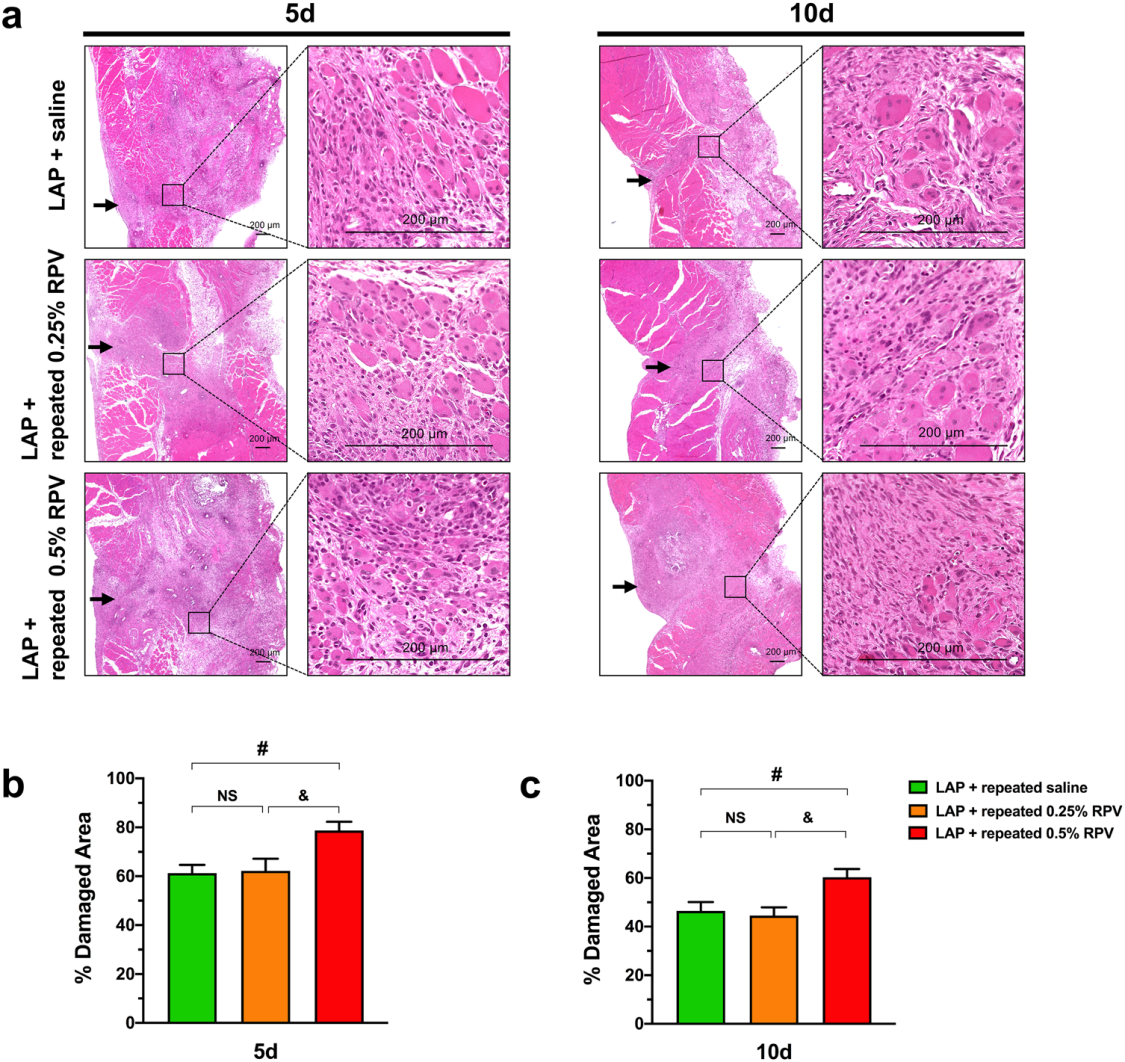
Damaged area in and around the incision site after repeated subfascial infiltration of saline or 0.25% ropivacaine or 0.5% ropivacaine. (**a**) Representative images of H&E-stained cross sections of the abdominal muscle tissues in and around the incision site at low magnification (left) and at high magnification (right) on days 5 and 10 after laparotomy. Black arrowheads show the incision line. Scale bar, 200 μm. Percents of damaged area in and around the incision site per area on (**b**) day 5 and (**c**) day 10. Data are presented as means ± SEM (n = 5 for each group) and were analyzed by one-way ANOVA for repeated measures with Tukey’s post hoc test. #*P* < 0.05 vs LAP + repeated saline group. Significant differences between the LAP + repeated 0.25% RPV group and LAP + repeated 0.5% RPV group was presented as &*P* < 0.05. See Supplementary Table S1 for details on statistics. RPV indicates ropivacaine; LAP, laparotomy; NS, no significant; H&E, hematoxylin and eosin; SEM, standard error of the mean; ANOVA, analysis of variance.

The percent of damaged area in the LAP + repeated 0.25% RPV group was not significantly different from that in the laparotomy + repeated administration of saline (LAP + repeated saline) group at day 5 or day 10 post-surgery. However, the percent of damaged area in the LAP + repeated 0.5% RPV group at day 5 and day 10 post-surgery were significantly larger than those in the LAP + repeated saline group (day 5, 79% ± 4% vs 61% ± 3%, *P* = 0.0253; day 10, 60% ± 3% vs 46% ± 4%, *P* = 0.0397) and LAP + repeated 0.25% RPV group (day 5, 79% ± 4% vs 62% ± 5%, *P* = 0.0341; day 10, 60% ± 3% vs 45% ± 3%, *P* = 0.0199) (Fig. 7a–c).

**Figure 8:**
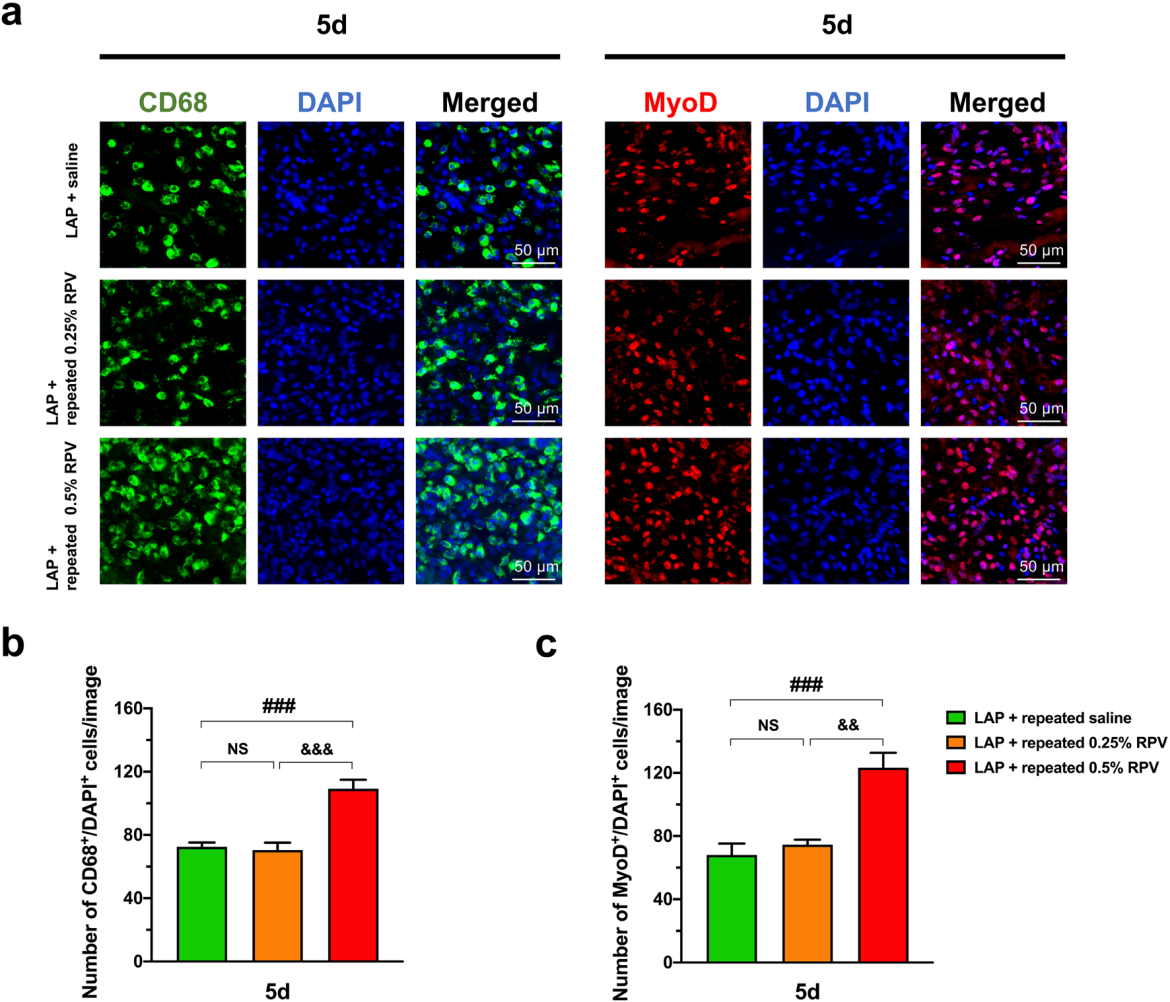
Infiltration of CD68-positive cells and expression of MyoD-positive cells in and around the incision site after repeated subfascial infiltration of saline or 0.25% ropivacaine or 0.5% ropivacaine. (**a**) Representative images of immunofluorescence staining of CD68 and MyoD in and around the incision site at high magnification on day 5 after laparotomy. Green, CD68; red, MyoD; blue, DAPI. Scale bar, 50 μm. (**b**) The number of CD68^+^/DAPI^+^ cells and (**c**) the number of MyoD^+^/DAPI^+^ cells in and around the incision site were counted on day 5 after laparotomy. Data are presented as means ± SEM (n = 5 for each group) and were analyzed by one-way ANOVA for repeated measures with Tukey’s post hoc test. ###*P* < 0.001 vs LAP + repeated saline group. Significant differences between the LAP + repeated 0.25% RPV group and LAP + repeated 0.5% RPV group was presented as &&*P* < 0.01 and &&&*P* < 0.001. See Supplementary Table S1 for details on statistics. RPV indicates ropivacaine; LAP, laparotomy; NS, no significant; CD68, a marker of macrophage infiltration; MyoD, a marker of satellite cell proliferation; DAPI, 4’,6-diamidino-2-phenylindole; SEM, standard error of the mean; ANOVA, analysis of variance.

As shown in Fig. 8, there were no significant differences in the number of CD68^+^/DAPI^+^ cells at day 5 post-surgery and number of MyoD^+^/DAPI^+^ cells at day 5 post-surgery between the LAP + repeated saline and LAP + repeated 0.25% RPV groups. In contrast, the number of CD68^+^/DAPI^+^ cells at day 5 post-surgery in the LAP + repeated 0.5% RPV group was significantly larger than that in the LAP + saline group (109 ± 6 vs 72 ± 3 per image, *P* = 0.0002) and LAP + 0.25% RPV group (109 ± 6 vs 70 ± 5 per image, *P* = 0.0001) (Fig. 8b). Similarly, the number of MyoD^+^/DAPI^+^ cells at day 5 post-surgery in the LAP + repeated 0.5% RPV group was also significantly larger than that in the LAP + saline group (123 ± 9 vs 68 ± 7 per image, *P* = 0.0004) and LAP + repeated 0.25% RPV group (123 ± 9 vs 75 ± 3 per image, *P* = 0.001) (Fig. 8c).

## Discussion

To the best of our knowledge, this is the first study showing the effects of a local anesthetic on damage, inflammation and regeneration in surgically incised abdominal muscles. The major findings of this study were as follows: (1) single and repeated subfascial infiltration of 0.25% ropivacaine did not augment abdominal muscle damage, macrophage infiltration and regeneration of muscle fibers after surgical incision, (2) a single subfascial infiltration of 0.5% ropivacaine did not induce injury in intact muscles without incision, but either single or repeated administration augmented damage of incised muscle tissues with increased inflammation, and (3) pain-related behaviors were inhibited by both 0.25% and 0.5% of ropivacaine for a short time after surgery, without a significant difference between the two groups.

The key strength of this study was that our experimental design reflected clinical conditions in a reliable and clinically relevant way. Ropivacaine is considered to be less likely to cause myotoxicity and systemic toxicity including neurotoxicity and cardiotoxicity than equal amounts of bupivacaine^7,9^. Various amounts (10–30 ml) and concentrations (0.2–0.75%) of ropivacaine has been used in a clinical setting^10,11^. In the current study, 100 μl of 0.25% ropivacaine (approximately 1.5 mg/kg) and 0.5% ropivacaine (approximately 3.0 mg/kg) were infiltrated below the fascia for a 2-cm-long incision in rats weighing approximately 200 g. These doses, which are far less than the dose of 10 mg/kg that has been reported to induce seizure activity in rats^9^, were able to reduce laparotomy-induced pain transiently assessed by RGS and ACT.

It has been shown that ropivacaine at concentrations above 0.5% injected intramuscularly has myotoxic effects on intact muscles^12^. In the current study, single subfascial infiltration of 0.5% ropivacaine below the fascia (not intramuscularly, Fig. 1c) of intact muscles without incision did not increase the muscle injury compared to that in sham surgery. In contrast, single subfascial infiltration of 0.5% ropivacaine augmented the damage in surgically incised muscles compared to the effects of saline and 0.25% ropivacaine. The results suggest that the myotoxic effects of local anesthetics vary depending on the state of skeletal muscles (intact or injured) and their concentrations. Disruption of cellular homeostasis via a complex cascade of calcium cytoplasmic efflux from the sarcoplasmic reticulum and alterations to mitochondrial bioenergetics may be considered as key mechanisms of local anesthetic-induced myotoxic effects in intact muscles^13,14^. Intramuscular extracellular matrix structures including the endomysium, perimysium and epimysium^15^ were probably injured when the muscles were surgically incised. Therefore, it is presumed that ropivacaine below the fascia easily reached incised muscle fibers along the incision site, resulting in augmentation of damage after laparotomy.

An inflammatory response is induced in the first stage of wound healing with neutrophils migrating into injured tissue firstly followed by migration of macrophages^16^. In this study, single dose administration of 0.5% ropivacaine increased infiltration of CD68-positive macrophages in and around the incisional site at both day 2 and day 5 post-surgery when compared to saline and 0.25% ropivacaine. It is likely that 0.5% ropivacaine augments muscle injury after laparotomy, leading to increases in the number of CD68-positive macrophages^17^. CD68-positve macrophages are not only involved in the inflammatory response, tissue destruction, and removal of necrotic tissue debris but are also necessary for progression of satellite cells from the proliferation stage to the differentiation stage^18^.

Satellite cells mediate muscle repair and regeneration after injury, which are essential processes in wound healing^19^. Upon muscle injury, quiescent satellite cells become activated to proliferate, followed by differentiation into myocytes^20^. MyoD, one of the myogenic transcription factors, is expressed early in the process of muscle regeneration, and its expression indicates the proliferation and differentiation of satellite cells^21,22^. In a study on compression-induced muscle injury in rats, expression of MyoD was found at 2 days after injury and its expression reached a maximum level at 4 days after injury^23^. In our study, single subfascial infiltration of 0.5% ropivacaine significantly increased the expression of MyoD in and around the surgical site at day 5 when compared to saline and 0.25% ropivacaine. It is presumed that infiltration of 0.5% ropivacaine may augment injury and inflammation, resulting in the proliferation and differentiation of more satellite cells rather than promotion of muscle regeneration.

Clinically, continuous wound infiltration with catheters placed below the muscle fascia is recommended in abdominal surgery as a part of multimodal analgesia to make up for the shortcoming of a short analgesic duration^1^. In our study, infiltration of 0.25% ropivacaine and 0.5% ropivacaine via a catheter transiently increased ACT, indicating that ropivacaine spread to the muscle wound area. No significant differences were noted between the LAP + repeated 0.25% RPV and LAP + repeated saline groups in the percent of damaged area, number of CD68-positive cells and MyoD expression, indicating that repeated infiltration of 0.25% ropivacaine does not affect the injury of incised muscles, infiltration of macrophages, and muscle regeneration. Similar to the results of single subfascial infiltration, repeated subfascial infiltration of 0.5% ropivacaine aggravated damage and inflammation of incised muscle tissues compared to the effects of saline and 0.25% ropivacaine.

Augmented damage and inflammatory responses in surgically incised muscles after subfascial infiltration of 0.5% ropivacaine observed in this study may be subclinical in most cases in clinical practice. Although extremely rare, suspected cases of local anesthetic-induced myotoxicity after continuous adductor canal block^14^ and after interscalene block^24^ have been reported. We cannot exclude the possibility that local anesthetics at high concentrations lead to further aggravation of damage in incised skeletal muscles. In our study, no significant differences were evident in the pain-related behaviors between 0.25% ropivacaine and 0.5% ropivacaine. Considering the balance between beneficial and harmful effects, administration of an unnecessarily high concentration of ropivacaine should be avoided. The optimal concentration and dosage of local anesthetics when administered near incised muscles need to be further investigated.

There were some limitations in this study. First, MyoD is involved in the process of activation, proliferation and early differentiation of satellite cells, while myogenin, another myogenic transcription factor at a later state of differentiation, also plays an important role in the terminal differentiation and formation of myotubes^19^. In the present study, MyoD-positive cells were evaluated at day 5, but the expression of myogenin was not analyzed. In other words, the effects of ropivacaine on the later phase of muscle regeneration were not investigated. Second, we demonstrated that single and repeated infiltration anesthesia of 0.5% ropivacaine enhanced damage of incised muscles at day 5 and day 10. However, how long the adverse effects last was not investigated. Morphological tissue changes in the intact muscles of animals receiving a single dose of 0.75% ropivacaine and continuous infusion of 0.375% ropivacaine for 6 h were detected at day 28^25^. The process of muscle regeneration is complex and takes time. Further work will be necessary to clarify the long-term effects of local anesthetics on the healing process of incisional muscle injury.

In conclusion, our results showed that single or repeated infiltration of 0.25% ropivacaine below the muscle fascia did not affect muscle injury, inflammatory responses, and muscle regeneration after laparotomy. However, the use of 0.5% ropivacaine near the surgically incised muscles may be a concern because of potentially increased damage in incised muscles.

## Materials and methods

### Animals

Adult male Sprague–Dawley rats (Japan SLC, Hamamatsu, Japan) weighing 180–240 g (aged 6–7 weeks) were used. All experimental procedures were approved by the Institutional Animal Care and Use Committee of Shinshu University School of Medicine (Approval No. 020028) and were in accordance with the Ethics Guidelines for Investigations of Experimental Pain in Conscious Animals as issued by the International Association for the Study of Pain^26^. This study was performed in accordance with ARRIVE guidelines. The rats were housed individually in plastic cages in a temperature-controlled room (22–24 °C) under a 12-h/12-h light/dark cycle. Food and water were available ad libitum during experimental sessions. All efforts were made to minimize animal suffering and to reduce the number of animals used in this study.

### Study design

#### Experiment 1: single dose administration

Rats were randomized into 4 groups: sham group (anesthesia only), laparotomy + saline (LAP + saline) group, laparotomy + 0.25% ropivacaine (LAP + 0.25% RPV) group and laparotomy + 0.5% ropivacaine (LAP + 0.5% RPV) group. Laparotomy without intervention (LAP-only) group and subfascial infiltration of 0.5% ropivacaine without abdominal muscles incision (0.5% RPV-only) group were set for histological and immunohistochemical analyses. Rats received the intervention of 100 μl of saline (0.9% NaCl), 0.25% ropivacaine or 0.5% ropivacaine infiltration at the end of laparotomy (Fig. 1a,c). Ropivacaine hydrochloride (R-283) was purchased from Sigma-Aldrich (Tokyo, Japan) and diluted with saline on the day of the experiment.

#### Experiment 2: repeated dose administration

Rats were randomized into 3 groups: laparotomy + repeated administration of saline (LAP + repeated saline) group, laparotomy + repeated administration of 0.25% ropivacaine (LAP + repeated 0.25% RPV) group and laparotomy + repeated administration of 0.5% ropivacaine (LAP + repeated 0.5% RPV) group. Rats received the intervention of 100 μl of saline, 0.25% ropivacaine or 0.5% ropivacaine infiltration at the end of laparotomy and day 1, day 2, day 3 and day 4 after laparotomy (Fig. 1b,d).

#### Laparotomy and Intervention

In experiment 1, laparotomy was performed as described in previous reports^27,28^. Briefly, under sevoflurane (3–4%) in 100% oxygen via a nose cone, a blade (#15) was used to make a 2.5-cm incision in abdominal skin diagonally 0.5 cm below and parallel to the lowest rib on the right side. A 2-cm-long incision was made through the fascia, abdominal muscles and peritoneum by using small blunt scissors. Approximately 10 cm of the small intestine was exteriorized and manipulated gently for 2 min. Abdominal wall muscles, including the external and internal oblique muscles and transversus abdominis muscle, and the parietal peritoneum were reapproximated with sterile, absorbable sutures (3–0 PDS*Plus Polydioxanone, ETHICON, USA) and then the fascia of the external oblique muscle was sutured at 5-mm intervals. As shown in Fig. 1c, subfascial infiltration of 100 μl of 0.25% ropivacaine, 0.5% ropivacaine or saline under visualization was done along the incisional line of the muscles slowly and carefully while avoiding intramuscular administration. Twenty-five μl of ropivacaine or saline was infiltrated by using a Hamilton syringe (Hamilton Company Inc., USA) at 5-mm intervals so that the drug would be able to infiltrate evenly over the entire 2-cm-long incision (totally, 100 μl of ropivacaine or saline being used in each animal). After confirmation of infiltration of the allocated drug, with no leakage to the outside of the fascia, the skin was closed using surgical sutures of 1–0 nylon.

In experiment 2, the same laparotomy as that in experiment 1 was performed except for placement of the catheter. After the abdominal wall muscles and parietal peritoneum had been reapproximated, a 15-cm-long multiorifice catheter (E19I-OE70, φ0.6 mm, Hakko Co., Ltd, Japan) with 15 side-holes evenly within 2 cm from the top was placed below the fascia of the external oblique muscle along the incisional line to repeatedly administer ropivacaine or saline, and the muscle fascia was closed and sutured to the catheter (Fig. 1d). Then 140 μl (the dead space in the catheter being approximately 40 μl) of 0.25% ropivacaine, 0.5% ropivacaine, or saline was injected via the catheter at a constant rate within 60 s to allow for diffusion followed by withdrawal of 40 μl of the allocated drug in the catheter. After confirmation of subfascial infiltration of the allocated drug, the catheter was tunneled subcutaneously to emerge at the posterior neck at which a 1-cm-long incision was made through the skin and then the skin was closed. The allocated drug was injected via the catheter at day 1, day 2, day 3 and day 4 after surgery.

#### Behavioral tests

Before beginning of the experiment, rats were acclimated for 2 days for habituation to the test environment. Behavioral tests were performed on the third day as baseline tests. The researcher performing the behavioral tests was blinded to the study treatment. Spontaneous pain and abdominal constriction threshold (ACT) were assessed by the Rat Grimace Scale (RGS)^29^ and von Frey filaments, respectively. Rats were placed individually in a clear plastic cage (14 × 10 × 15 cm) on a stainless mesh floor (openings, 8 × 8 mm) to be acclimated to the test environment for 10 min.

Ten still front-view images including all facial features were cropped at 3-min intervals from each 30-min video recording for each rat. Four separate action units including orbital tightening, nose/cheek fluttering, ear position, and whisker bunching were scored by using a 3-point scale (no pain = 0, moderate pain = 1, obvious pain = 2) in each image. The final RGS score for each rat was calculated by taking the average score of the four RGS action units from 10 images.

ACT was measured through a series of calibrated von Frey filaments of 2, 4, 6, 8, 10, 15 and 26 g (Stoelting, Wood Dale, IL, USA)^30^. The von Frey filaments starting with the least force of 2 g were applied once for 3 s to the right abdominal skin surface (near the wound line) with increasing stiffness sequentially until an abdominal constriction response was evoked. The cutoff to prevent tissue damage was determined to be 26 g, which was recorded even if there was no response to the force of 26 g. Three tests were carried out at 3-min intervals and the lowest force of the filament from the three tests was considered as the ACT.

#### Histology and immunofluorescence

Rats were deeply anesthetized with sevoflurane and perfused transcardially with 4% paraformaldehyde (PFA) in 0.1 M phosphate buffer. Muscle tissues (1 × 2 cm) with wounds in the center were fixed in 4% PFA.

For cross-sectional histology, the muscle tissues were embedded in paraffin. Four-μm-thick cross sections were cut and stained with hematoxylin and eosin (H&E). Muscle fibers with peripherally or centrally located nuclei were considered to be normal or regenerating fibers, respectively. An area where there were no normal or regenerating fibers was defined as the damaged area^31^. Then the proportion of the damaged area of 500 μm in length and 2600 μm in width, with wounds in the center, was calculated (Supplementary Fig. S1). Four selected microscopic fields from two random sections in each rat were measured and analyzed by observers who were blinded to the experimental groups.

For immunofluorescence, muscle tissues were postfixed in 4% PFA for 2 h and placed in 30% sucrose overnight at 4 °C. Sections (20 μm thick) were incubated with the following primary antibodies at 4 °C overnight: CD68 (rabbit, 1:1000, ab125212, Abcam), a marker of macrophage infiltration to evaluate tissue inflammation^32,33^, and MyoD (mouse, 1:100, sc-377460, Santa Cruz Biotechnology), a marker of satellite cells starting to proliferate and differentiate to evaluate muscle tissue regeneration^21,22^. Sections were incubated at room temperature for 90 min with secondary antibodies labeled with Alexa Fluor 488 goat anti-rabbit (1:500, ab150077, Abcam) or Alexa Fluor 594 goat anti-mouse (1:500, ab150116, Abcam) followed by nuclei staining with 4’,6-diamidino-2-phenylindole (DAPI; Vector Laboratories, Burlingame, California). Fluorescent images were obtained with a KEYENCE model BZ-X810 All-in-One Fluorescence Microscope (Osaka, Japan). Thirty images (362 × 271 μm) from 3 randomly selected sections in each rat were obtained and analyzed. The numbers of CD68^+^/DAPI^+^ and MyoD^+^/DAPI^+^ cells were counted by observers who were blinded to the experimental groups using Image J software (National Institutes of Health, Bethesda, Maryland) and the mean of the three sections was calculated.

#### Statistical Analysis

For continuous data, the normal distribution of values was determined by the Shapiro–Wilk test. The data are presented as means ± standard error of the mean (SEM), and GraphPad Prism 8 (GraphPad Software, San Diego, California) was used for statistical analyses. The sample size was determined on the basis of previous studies^34,35^. Data for RGS scores were compared by two-way analysis of variance (ANOVA) for repeated measures with Tukey’s post hoc test. For ACT, the Kruskal–Wallis test for between-group comparisons followed by Dunnett’s post hoc test was used, and presented as the median with first and third quartiles. The percent of damaged area and the numbers of CD68^+^/DAPI^+^ and MyoD^+^/DAPI^+^ cells were analyzed by one-way ANOVA for repeated measures with Tukey’s post hoc test. *P* < 0.05 was considered statistically significant.

## Supplementary Information

Supplementary Information.

## Data Availability

The datasets generated and/or analyzed during the present study are available from the corresponding author upon reasonable request.
