# Supplementary material for: Subfascial infiltration of 0.5% ropivacaine, but not 0.25% ropivacaine, exacerbates damage and inflammation in surgically incised abdominal muscles of rats
Source: Sci Rep. 2022 Jun 7;12:9409. doi: 10.1038/s41598-022-13628-w (PMC9174254; doi:10.1038/s41598-022-13628-w)
Supplement: Supplementary file 1 — Supplementary Information. [file 41598_2022_13628_MOESM1_ESM.pdf]

Subfascial infiltration of 0.5% ropivacaine, but not 0.25% ropivacaine, exacerbates damage and inflammation in surgically incised abdominal muscles of rats.

Dandan Shen[1], Yuki Sugiyama[1], Kumiko Ishida[1], Satoshi Fuseya[1], Takashi Ishida[1], Mikito Kawamata[1], Satoshi Tanaka[1,*]

[1]Department of Anesthesiology and Resuscitology, Shinshu University School of Medicine, Matsumoto City, Nagano 390-8621, Japan

Dandan Shen[1]: shendandan6689@gmail.com
Yuki Sugiyama[1]: ysugiyama@shinshu-u.ac.jp
Kumiko Ishida[1]: kumiko_m@shinshu-u.ac.jp
Satoshi Fuseya[1]: sfuseya@shinshu-u.ac.jp
Takashi Ishida[1]: tisd@shinshu-u.ac.jp
Mikito Kawamata[1]: kawamata@shinshu-u.ac.jp
Satoshi Tanaka[1,*]: s_tanaka@shinshu-u.ac.jp

Address correspondence to:
Satoshi Tanaka,
Department of Anesthesiology and Resuscitology, Shinshu University School of Medicine, Matsumoto City, Nagano 390-8621, Japan.
Tel.: +81 263 37 2670
Fax: +81 263 35 2734
E-mail address: s_tanaka@shinshu-u.ac.jp

Supplementary Figure S1.

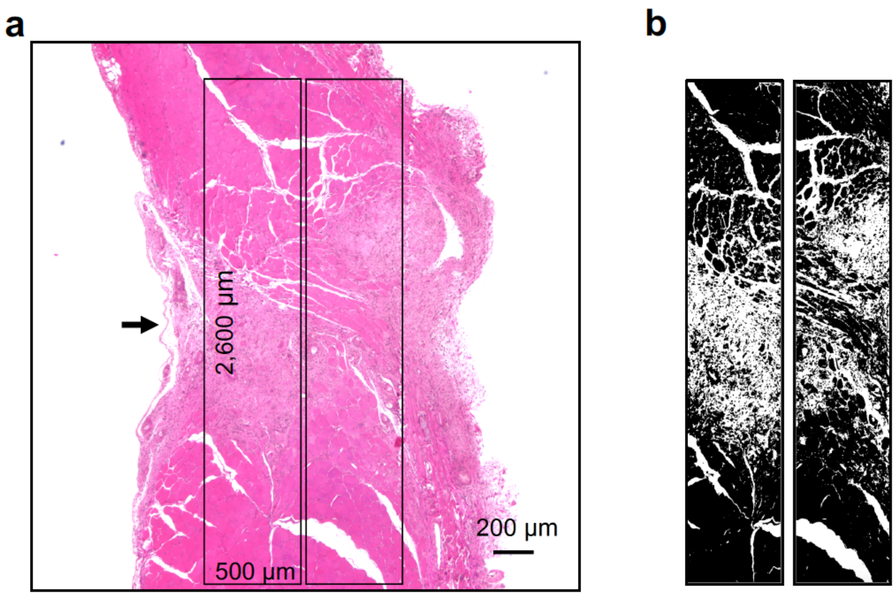

Supplementary Figure S1. An example of damage area analysis for H&E staining.

(a) An area of 500 µm in length and 2,600 µm in width with wounds in the center is framed. Scale bar, 200 µm. An area where there were no normal or regenerating fibers was defined as the damaged area. (b) The proportion of the damaged area (white area) in the 500 µm × 2,600 µm rectangle was calculated by using Image J software (National Institutes of Health, Bethesda, Maryland). Four selected microscopic fields from two random sections in each rat were measured and analyzed. A black arrowhead shows the incision line. H&E indicates hematoxylin and eosin.

Supplementary Figure S2.

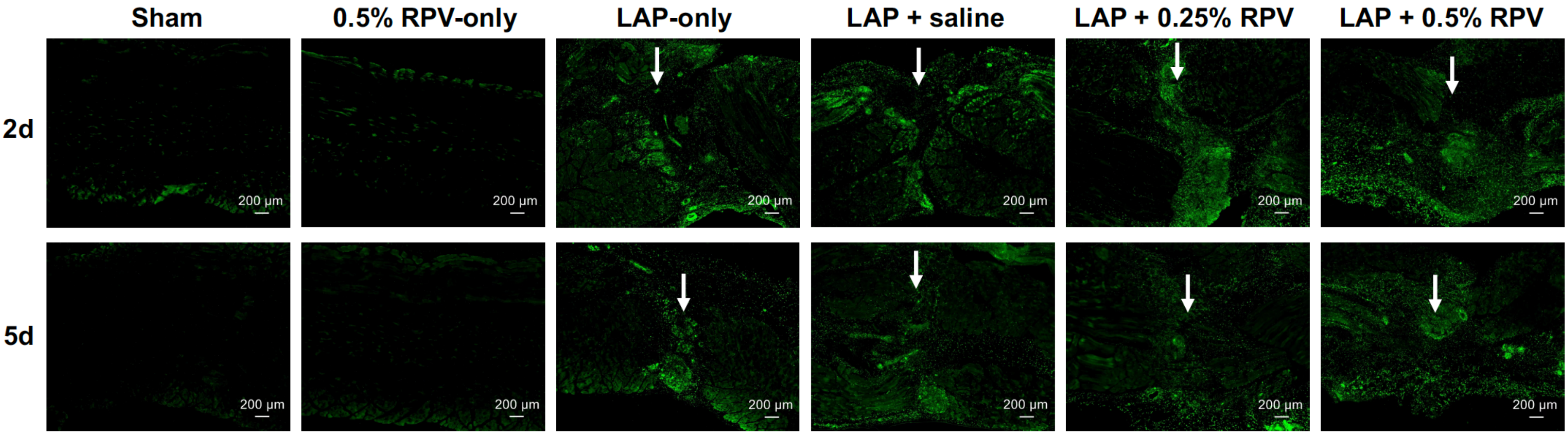

Supplementary Figure S2. Infiltration of CD68-positive cells in and around the incision site at low magnification after single subfascial infiltration of saline or 0.25% ropivacaine or 0.5% ropivacaine.

Typical examples of the distribution of CD68-positive cells in the entire field at low magnification at 2 days and 5 days after laparotomy. The distribution of CD68-positive cells was found in and around the incision site. White arrowheads show the incision line. Green, CD68. Scale bar, 200 μm. RPV indicates ropivacaine; LAP, laparotomy; CD68, a marker of macrophage infiltration.

Supplementary Table S1. The details of statistics used in this study

Figure	Panel	Number of sample	Test used	F and p value	Post hoc test	95% confidence interval [CI] and comparison significance		
2a	RGS Score	Sham = 6,<br>LAP + saline = 6,<br>LAP + 0.25% RPV = 6,<br>LAP + 0.5% RPV = 6	Two-way ANOVA assessed by repeated measures	Time: F (4.353, 87.07) = 46.65, p < 0.0001<br>Group: F (3, 20) = 46.90, p < 0.0001<br>Interaction: F (24, 160) = 5.971, p < 0.0001	Tukey's multiple comparison	Sham vs LAP + saline (baseline)	95%[CI], -0.2002 to 0.1169	p = 0.8396, ns
						Sham vs LAP + 0.25% RPV (baseline)	95%[CI], -0.07085 to 0.1292	p = 0.7781, ns
						Sham vs LAP + 0.5% RPV (baseline)	95%[CI], -0.1727 to 0.1311	p = 0.9718, ns
						LAP + saline vs LAP + 0.25% RPV (baseline)	95%[CI], -0.08230 to 0.2240	p = 0.4442, ns
						LAP + saline vs LAP + 0.5% RPV (baseline)	95%[CI], -0.1571 to 0.1987	p = 0.9833, ns
						LAP + 0.25% RPV vs LAP + 0.5% RPV (baseline)	95%[CI], -0.1952 to 0.09523	p = 0.6572, ns
						Sham vs LAP + saline (2h)	95%[CI], -1.097 to -0.2359	p = 0.0060
						Sham vs LAP + 0.25% RPV (2h)	95%[CI], -0.3785 to 0.02854	p = 0.0976, ns
						Sham vs LAP + 0.5% RPV (2h)	95%[CI], -0.3827 to 0.03266	p = 0.1062, ns
						LAP + saline vs LAP + 0.25% RPV (2h)	95%[CI], 0.06137 to 0.9220	p = 0.0284
						LAP + saline vs LAP + 0.5% RPV (2h)	95%[CI], 0.06142 to 0.9219	p = 0.0282
						LAP + 0.25% RPV vs LAP + 0.5% RPV (2h)	95%[CI], -0.1873 to 0.1873	p > 0.9999, ns
						Sham vs LAP + saline (6h)	95%[CI], -0.8323 to -0.4510	p < 0.0001
						Sham vs LAP + 0.25% RPV (6h)	95%[CI], -0.6561 to -0.1439	p = 0.0047
						Sham vs LAP + 0.5% RPV (6h)	95%[CI], -0.6664 to -0.1669	p = 0.0031
						LAP + saline vs LAP + 0.25% RPV (6h)	95%[CI], -0.02296 to 0.5063	p = 0.0750, ns
						LAP + saline vs LAP + 0.5% RPV (6h)	95%[CI], -0.03390 to 0.4839	p = 0.0924, ns
						LAP + 0.25% RPV vs LAP + 0.5% RPV (6h)	95%[CI], -0.3134 to 0.2800	p = 0.9981, ns
						Sham vs LAP + saline (1d)	95%[CI], -0.6643 to -0.4773	p < 0.0001
						Sham vs LAP + 0.25% RPV (1d)	95%[CI], -0.5912 to -0.2255	p = 0.0007
						Sham vs LAP + 0.5% RPV (1d)	95%[CI], -0.7050 to -0.07835	p = 0.0199
						LAP + saline vs LAP + 0.25% RPV (1d)	95%[CI], -0.02026 to 0.3453	p = 0.0801, ns
						LAP + saline vs LAP + 0.5% RPV (1d)	95%[CI], -0.1342 to 0.4926	p = 0.2832, ns
						LAP + 0.25% RPV vs LAP + 0.5% RPV (1d)	95%[CI], -0.3018 to 0.3351	p = 0.9982, ns
						Sham vs LAP + saline (2d)	95%[CI], -0.7077 to -0.3506	p = 0.0001
						Sham vs LAP + 0.25% RPV (2d)	95%[CI], -0.7671 to -0.1746	p = 0.0066
						Sham vs LAP + 0.5% RPV (2d)	95%[CI], -0.6765 to -0.1652	p = 0.0053

Figure	Panel	Number of sample	Test used	F and p value	Post hoc test	95% confidence interval [CI] and comparison significance		
2a	RGS Score	Sham = 6,<br>LAP + saline = 6,<br>LAP + 0.25% RPV = 6,<br>LAP + 0.5% RPV = 6	Two-way ANOVA assessed by repeated measures	Time: $F (4.353, 87.07) = 46.65, p < 0.0001$<br>Group: $F (3, 20) = 46.90, p < 0.0001$<br>Interaction: $F (24, 160) = 5.971, p < 0.0001$	Tukey's multiple comparison	LAP + saline vs LAP + 0.25% RPV (2d)	95%[CI], -0.2440 to 0.3607	p = 0.9249, ns
						LAP + saline vs LAP + 0.5% RPV (2d)	95%[CI], -0.1598 to 0.3765	p = 0.6064, ns
						LAP + 0.25% RPV vs LAP + 0.5% RPV (2d)	95%[CI], -0.2801 to 0.3801	p = 0.9650, ns
						Sham vs LAP + saline (3d)	95%[CI], -0.5129 to -0.1371	p = 0.0018
						Sham vs LAP + 0.25% RPV (3d)	95%[CI], -0.4603 to -0.1397	p = 0.0009
						Sham vs LAP + 0.5% RPV (3d)	95%[CI], -0.5575 to -0.01746	p = 0.0376
						LAP + saline vs LAP + 0.25% RPV (3d)	95%[CI], -0.1561 to 0.2061	p = 0.9720, ns
						LAP + saline vs LAP + 0.5% RPV (3d)	95%[CI], -0.2383 to 0.3133	p = 0.9718, ns
						LAP + 0.25% RPV vs LAP + 0.5% RPV (3d)	95%[CI], -0.2556 to 0.2806	p = 0.9986, ns
						Sham vs LAP + saline (5d)	95%[CI], -0.5061 to -0.1606	p = 0.0010
						Sham vs LAP + 0.25% RPV (5d)	95%[CI], -0.3889 to -0.07772	p = 0.0049
						Sham vs LAP + 0.5% RPV (5d)	95%[CI], -0.4025 to -0.08913	p = 0.0037
						LAP + saline vs LAP + 0.25% RPV (5d)	95%[CI], -0.08588 to 0.2859	p = 0.3963, ns
						LAP + saline vs LAP + 0.5% RPV (5d)	95%[CI], -0.09908 to 0.2741	p = 0.5058, ns
						LAP + 0.25% RPV vs LAP + 0.5% RPV (5d)	95%[CI], -0.1855 to 0.1605	p = 0.9959, ns
						Sham vs LAP + saline (7d)	95%[CI], -0.4713 to 0.1046	p = 0.2369, ns
						Sham vs LAP + 0.25% RPV (7d)	95%[CI], -0.2077 to 0.07434	p = 0.4993, ns
						Sham vs LAP + 0.5% RPV (7d)	95%[CI], -0.2969 to 0.01355	p = 0.0763, ns
						LAP + saline vs LAP + 0.25% RPV (7d)	95%[CI], -0.1708 to 0.4042	p = 0.5528, ns
						LAP + saline vs LAP + 0.5% RPV (7d)	95%[CI], -0.2469 to 0.3303	p = 0.9620, ns
						LAP + 0.25% RPV vs LAP + 0.5% RPV (7d)	95%[CI], -0.2225 to 0.07252	p = 0.4391, ns
						Sham vs LAP + saline (10d)	95%[CI], -0.1802 to 0.08020	p = 0.6540, ns
						Sham vs LAP + 0.25% RPV (10d)	95%[CI], -0.1229 to 0.1313	p = 0.9996, ns
						Sham vs LAP + 0.5% RPV (10d)	95%[CI], -0.07821 to 0.1615	p = 0.7063, ns
						LAP + saline vs LAP + 0.25% RPV (10d)	95%[CI], -0.06617 to 0.1745	p = 0.5392, ns
						LAP + saline vs LAP + 0.5% RPV (10d)	95%[CI], -0.02019 to 0.2035	p = 0.1173, ns
						LAP + 0.25% RPV vs LAP + 0.5% RPV (10d)	95%[CI], -0.06976 to 0.1448	p = 0.7106, ns

Figure	Panel	Number of sample	Test used	F and p value	Post hoc test	95% confidence interval [CI] and comparison significance		
2b	Abdominal Constriction Threshold	Sham = 6,<br>LAP + saline = 6,<br>LAP + 0.25% RPV = 6,<br>LAP + 0.5% RPV = 6	Kruskal-Wallis test		Dunnett's multiple comparison	Sham vs LAP + saline (baseline)		p > 0.9999, ns
						Sham vs LAP + 0.25% RPV (baseline)		p > 0.9999, ns
						Sham vs LAP + 0.5% RPV (baseline)		p = 0.2624, ns
						LAP + saline vs LAP + 0.25% RPV (baseline)		p > 0.9999, ns
						LAP + saline vs LAP + 0.5% RPV (baseline)		p > 0.9999, ns
						LAP + 0.25% RPV vs LAP + 0.5% RPV (baseline)		p = 0.2624, ns
						Sham vs LAP + saline (2h)		p = 0.0004
						Sham vs LAP + 0.25% RPV (2h)		p > 0.9999, ns
						Sham vs LAP + 0.5% RPV (2h)		p > 0.9999, ns
						LAP + saline vs LAP + 0.25% RPV (2h)		p = 0.0024
						LAP + saline vs LAP + 0.5% RPV (2h)		p = 0.0004
						LAP + 0.25% RPV vs LAP + 0.5% RPV (2h)		p > 0.9999, ns
						Sham vs LAP + saline (6h)		p = 0.0004
						Sham vs LAP + 0.25% RPV (6h)		p = 0.0202
						Sham vs LAP + 0.5% RPV (6h)		p = 0.0417
						LAP + saline vs LAP + 0.25% RPV (6h)		p = 0.7808, ns
						LAP + saline vs LAP + 0.5% RPV (6h)		p = 0.5065, ns
						LAP + 0.25% RPV vs LAP + 0.5% RPV (6h)		p > 0.9999, ns
						Sham vs LAP + saline (1d)		p = 0.0011
						Sham vs LAP + 0.25% RPV (1d)		p = 0.0056
						Sham vs LAP + 0.5% RPV (1d)		p = 0.0467
						LAP + saline vs LAP + 0.25% RPV (1d)		p > 0.9999, ns
						LAP + saline vs LAP + 0.5% RPV (1d)		p = 0.7681, ns
						LAP + 0.25% RPV vs LAP + 0.5% RPV (1d)		p > 0.9999, ns
						Sham vs LAP + saline (2d)		p = 0.0021
						Sham vs LAP + 0.25% RPV (2d)		p = 0.0084
						Sham vs LAP + 0.5% RPV (2d)		p = 0.0165

Figure	Panel	Number of sample	Test used	F and p value	Post hoc test	95% confidence interval [CI] and comparison significance		
2b	Abdominal Constriction Threshold	Sham = 6,<br>LAP + saline = 6,<br>LAP + 0.25% RPV = 6,<br>LAP + 0.5% RPV = 6	Kruskal-Wallis test		Dunnett’s multiple comparison	LAP + saline vs LAP + 0.25% RPV (2d)		p > 0.9999, ns
						LAP + saline vs LAP + 0.5% RPV (2d)		p > 0.9999, ns
						LAP + 0.25% RPV vs LAP + 0.5% RPV (2d)		p > 0.9999, ns
						Sham vs LAP + saline (3d)		p = 0.0068
						Sham vs LAP + 0.25% RPV (3d)		p = 0.0103
						Sham vs LAP + 0.5% RPV (3d)		p = 0.0323
						LAP + saline vs LAP + 0.25% RPV (3d)		p > 0.9999, ns
						LAP + saline vs LAP + 0.5% RPV (3d)		p > 0.9999, ns
						LAP + 0.25% RPV vs LAP + 0.5% RPV (3d)		p > 0.9999, ns
						Sham vs LAP + saline (5d)		p = 0.0070
						Sham vs LAP + 0.25% RPV (5d)		p = 0.1610, ns
						Sham vs LAP + 0.5% RPV (5d)		p = 0.0773, ns
						LAP + saline vs LAP + 0.25% RPV (5d)		p = 0.7948, ns
						LAP + saline vs LAP + 0.5% RPV (5d)		p > 0.9999, ns
						LAP + 0.25% RPV vs LAP + 0.5% RPV (5d)		p > 0.9999, ns
						Sham vs LAP + saline (7d)		p = 0.0170
						Sham vs LAP + 0.25% RPV (7d)		p = 0.0731, ns
						Sham vs LAP + 0.5% RPV (7d)		p = 0.0863, ns
						LAP + saline vs LAP + 0.25% RPV (7d)		p > 0.9999, ns
						LAP + saline vs LAP + 0.5% RPV (7d)		p > 0.9999, ns
						LAP + 0.25% RPV vs LAP + 0.5% RPV (7d)		p > 0.9999, ns
						Sham vs LAP + saline (10d)		p = 0.1264, ns
						Sham vs LAP + 0.25% RPV (10d)		p = 0.2942, ns
						Sham vs LAP + 0.5% RPV (10d)		p = 0.4686, ns
						LAP + saline vs LAP + 0.25% RPV (10d)		p > 0.9999, ns
						LAP + saline vs LAP + 0.5% RPV (10d)		p > 0.9999, ns
						LAP + 0.25% RPV vs LAP + 0.5% RPV (10d)		p > 0.9999, ns

Figure	Panel	Number of sample	Test used	F and p value	Post hoc test	95% confidence interval [CI] and comparison significance		
3b	% Damaged Area 5d	Sham = 4,<br>0.5% RPV-only = 4,<br>LAP-only = 5,<br>LAP + saline = 5,<br>LAP + 0.25% RPV = 5,<br>LAP + 0.5% RPV = 5	One-way ANOVA	Treatment: F (5, 22) = 52.50, p < 0.0001	Tukey's multiple comparison	Sham vs 0.5% RPV-only	95%[CI], -15.34 to 11.69	p = 0.9981, ns
						Sham vs LAP-only	95%[CI], -46.42 to -20.77	p < 0.0001
						Sham vs LAP + saline	95%[CI], -49.35 to -23.71	p < 0.0001
						Sham vs LAP + 0.25% RPV	95%[CI], -45.95 to -20.30	p < 0.0001
						Sham vs LAP + 0.5% RPV	95%[CI], -66.74 to -41.09	p < 0.0001
						LAP + saline vs 0.5% RPV-only	95%[CI], -47.53 to -21.88	p < 0.0001
						LAP + saline vs LAP-only	95%[CI], -15.02 to 9.155	p = 0.9721, ns
						LAP + saline vs LAP + 0.25% RPV	95%[CI], -8.685 to 15.49	p = 0.9481, ns
						LAP + saline vs LAP + 0.5% RPV	95%[CI], -29.47 to -5.295	p = 0.0023
						LAP + 0.25% RPV vs LAP + 0.5% RPV	95%[CI], -32.88 to -8.699	p = 0.0003
3c	% Damaged Area 10d	Sham = 4,<br>0.5% RPV-only = 4,<br>LAP-only = 5,<br>LAP + saline = 5,<br>LAP + 0.25% RPV = 5,<br>LAP + 0.5% RPV = 5	One-way ANOVA	Treatment: F (5, 22) = 35.69, p < 0.0001	Tukey's multiple comparison	Sham vs 0.5% RPV-only	95%[CI], -14.49 to 7.524	p = 0.9177, ns
						Sham vs LAP-only	95%[CI], -35.54 to -14.66	p < 0.0001
						Sham vs LAP + saline	95%[CI], -32.76 to -11.88	p < 0.0001
						Sham vs LAP + 0.25% RPV	95%[CI], -34.96 to -14.08	p < 0.0001
						Sham vs LAP + 0.5% RPV	95%[CI], -47.79 to -26.91	p < 0.0001
						LAP + saline vs 0.5% RPV-only	95%[CI], -29.28 to -8.393	p = 0.0002
						LAP + saline vs LAP-only	95%[CI], -7.060 to 12.63	p = 0.9472, ns
						LAP + saline vs LAP + 0.25% RPV	95%[CI], -12.05 to 7.642	p = 0.9804, ns
						LAP + saline vs LAP + 0.5% RPV	95%[CI], -24.88 to -5.190	p = 0.0012
						LAP + 0.25% RPV vs LAP + 0.5% RPV	95%[CI], -22.68 to -2.988	p = 0.0061
4b	Number of CD68+/DAPI+ cells/image 2d	Sham = 4,<br>0.5% RPV-only = 4,<br>LAP-only = 5,<br>LAP + saline = 5,<br>LAP + 0.25% RPV = 5,<br>LAP + 0.5% RPV = 5	One-way ANOVA	Treatment: F (5, 22) = 104.7, p < 0.0001	Tukey's multiple comparison	Sham vs 0.5% RPV-only	95%[CI], -24.96 to 25.41	p > 0.9999, ns
						Sham vs LAP-only	95%[CI], -119.0 to -71.25	p < 0.0001
						Sham vs LAP + saline	95%[CI], -120.3 to -72.55	p < 0.0001
						Sham vs LAP + 0.25% RPV	95%[CI], -123.7 to -75.90	p < 0.0001
						Sham vs LAP + 0.5% RPV	95%[CI], -155.3 to -107.6	p < 0.0001
						LAP + saline vs 0.5% RPV-only	95%[CI], -120.6 to -72.78	p < 0.0001
						LAP + saline vs LAP-only	95%[CI], -23.83 to 21.23	p > 0.9999, ns
						LAP + saline vs LAP + 0.25% RPV	95%[CI], -25.88 to 19.17	p = 0.9970, ns
						LAP + saline vs LAP + 0.5% RPV	95%[CI], -57.54 to -12.49	p = 0.0010
						LAP + 0.25% RPV vs LAP + 0.5% RPV	95%[CI], -54.19 to -9.134	p = 0.0029

Figure	Panel	Number of sample	Test used	F and p value	Post hoc test	95% confidence interval [CI] and comparison significance		
4c	Number of $CD68^{+}$/$DAPI^{+}$ cells/image 5d	Sham = 4,<br>0.5% RPV-only = 4,<br>LAP-only = 5,<br>LAP + saline = 5,<br>LAP + 0.25% RPV = 5,<br>LAP + 0.5% RPV = 5	One-way ANOVA	Treatment: F (5, 22) = 195.6, p < 0.0001	Tukey's multiple comparison	Sham vs 0.5% RPV-only	95%[CI], -12.57 to 13.04	p > 0.9999, ns
						Sham vs LAP-only	95%[CI], -76.00 to -51.70	p < 0.0001
						Sham vs LAP + saline	95%[CI], -73.50 to -49.20	p < 0.0001
						Sham vs LAP + 0.25% RPV	95%[CI], -80.41 to -56.11	p < 0.0001
						Sham vs LAP + 0.5% RPV	95%[CI], -106.3 to -81.99	p < 0.0001
						LAP + saline vs 0.5% RPV-only	95%[CI], -73.73 to -49.43	p < 0.0001
						LAP + saline vs LAP-only	95%[CI], -8.956 to 13.95	p = 0.9824, ns
						LAP + saline vs LAP + 0.25% RPV	95%[CI], -18.37 to 4.542	p = 0.4395, ns
						LAP + saline vs LAP + 0.5% RPV	95%[CI], -44.25 to -21.34	p < 0.0001
						LAP + 0.25% RPV vs LAP + 0.5% RPV	95%[CI], -37.33 to -14.43	p < 0.0001
5b	Number of $MyoD^{+}$/$DAPI^{+}$ cells/image 5d	Sham = 4,<br>0.5% RPV-only = 4,<br>LAP-only = 5,<br>LAP + saline = 5,<br>LAP + 0.25% RPV = 5,<br>LAP + 0.5% RPV = 5	One-way ANOVA	Treatment: F (5, 22) = 84.81, p < 0.0001	Tukey's multiple comparison	Sham vs 0.5% RPV-only	95%[CI], -19.28 to 23.43	p = 0.9996, ns
						Sham vs LAP-only	95%[CI], -73.81 to -33.29	p < 0.0001
						Sham vs LAP + saline	95%[CI], -72.21 to -31.69	p < 0.0001
						Sham vs LAP + 0.25% RPV	95%[CI], -80.47 to -39.95	p < 0.0001
						Sham vs LAP + 0.5% RPV	95%[CI], -131.0 to -90.45	p < 0.0001
						LAP + saline vs 0.5% RPV-only	95%[CI], -74.29 to -33.77	p < 0.0001
						LAP + saline vs LAP-only	95%[CI], -17.51 to 20.69	p = 0.9998, ns
						LAP + saline vs LAP + 0.25% RPV	95%[CI], -27.36 to 10.84	p = 0.7566, ns
						LAP + saline vs LAP + 0.5% RPV	95%[CI], -77.86 to -39.66	p < 0.0001
						LAP + 0.25% RPV vs LAP + 0.5% RPV	95%[CI], -69.60 to -31.40	p < 0.0001
6	Abdominal Constriction Threshold	LAP + repeated saline = 6,<br>LAP + repeated 0.25% RPV = 6,<br>LAP + repeated 0.5% RPV = 6	Kruskal-Wallis test		Dunnett's multiple comparison	LAP + repeated saline vs LAP + repeated 0.25% RPV (baseline)		p = 0.9996, ns
						LAP + repeated saline vs LAP + repeated 0.5% RPV (baseline)		p > 0.9999, ns
						LAP + repeated 0.25% RPV vs LAP + repeated 0.5% RPV (baseline)		p = 0.9996, ns
						LAP + repeated saline vs LAP + repeated 0.25% RPV (2h)		p = 0.0059
						LAP + repeated saline vs LAP + repeated 0.5% RPV (2h)		p = 0.0018
						LAP + repeated 0.25% RPV vs LAP + repeated 0.5% RPV (2h)		p > 0.9999, ns
						LAP + repeated saline vs LAP + repeated 0.25% RPV (6h)		p > 0.9999, ns
						LAP + repeated saline vs LAP + repeated 0.5% RPV (6h)		p = 0.3406, ns
						LAP + repeated 0.25% RPV vs LAP + repeated 0.5% RPV (6h)		p = 0.8811, ns

Figure	Panel	Number of sample	Test used	F and p value	Post hoc test	95% confidence interval [CI] and comparison significance		
6	Abdominal Constriction Threshold	LAP + repeated saline = 6, LAP + repeated 0.25% RPV = 6, LAP + repeated 0.5% RPV = 6	Kruskal-Wallis test		Dunnett's multiple comparison	LAP + repeated saline vs LAP + repeated 0.25% RPV (pre 1d)		p = 0.2621, ns
						LAP + repeated saline vs LAP + repeated 0.5% RPV (pre 1d)		p = 0.5396, ns
						LAP + repeated 0.25% RPV vs LAP + repeated 0.5% RPV (pre 1d)		p > 0.9999, ns
						LAP + repeated saline vs LAP + repeated 0.25% RPV (post 1d)		p = 0.0056
						LAP + repeated saline vs LAP + repeated 0.5% RPV (post 1d)		p = 0.0017
						LAP + repeated 0.25% RPV vs LAP + repeated 0.5% RPV (post 1d)		p > 0.9999, ns
						LAP + repeated saline vs LAP + repeated 0.25% RPV (pre 2d)		p > 0.9999, ns
						LAP + repeated saline vs LAP + repeated 0.5% RPV (pre 2d)		p > 0.9999, ns
						LAP + repeated 0.25% RPV vs LAP + repeated 0.5% RPV (pre 2d)		p > 0.9999, ns
						LAP + repeated saline vs LAP + repeated 0.25% RPV (post 2d)		p = 0.0061
						LAP + repeated saline vs LAP + repeated 0.5% RPV (post 2d)		p = 0.0019
						LAP + repeated 0.25% RPV vs LAP + repeated 0.5% RPV (post 2d)		p > 0.9999, ns
						LAP + repeated saline vs LAP + repeated 0.25% RPV (pre 3d)		p = 0.8205, ns
						LAP + repeated saline vs LAP + repeated 0.5% RPV (pre 3d)		p = 0.5531, ns
						LAP + repeated 0.25% RPV vs LAP + repeated 0.5% RPV (pre 3d)		p = 0.1119, ns
						LAP + repeated saline vs LAP + repeated 0.25% RPV (post 3d)		p = 0.0061
						LAP + repeated saline vs LAP + repeated 0.5% RPV (post 3d)		p = 0.0019
						LAP + repeated 0.25% RPV vs LAP + repeated 0.5% RPV (post 3d)		p > 0.9999, ns
						LAP + repeated saline vs LAP + repeated 0.25% RPV (5d)		p > 0.9999, ns
						LAP + repeated saline vs LAP + repeated 0.5% RPV (5d)		p > 0.9999, ns
						LAP + repeated 0.25% RPV vs LAP + repeated 0.5% RPV (5d)		p > 0.9999, ns
						LAP + repeated saline vs LAP + repeated 0.25% RPV (7d)		p = 0.1369, ns
						LAP + repeated saline vs LAP + repeated 0.5% RPV (7d)		p = 0.1369, ns
						LAP + repeated 0.25% RPV vs LAP + repeated 0.5% RPV (7d)		p > 0.9999, ns

Figure	Panel	Number of sample	Test used	F and p value	Post hoc test	95% confidence interval [CI] and comparison significance		
6	Abdominal Constriction Threshold	LAP + repeated saline = 6, LAP + repeated 0.25% RPV = 6, LAP + repeated 0.5% RPV = 6	Kruskal-Wallis test		Dunnett's multiple comparison	LAP + repeated saline vs LAP + repeated 0.25% RPV (10d)		$p > 0.9999$, ns
						LAP + repeated saline vs LAP + repeated 0.5% RPV (10d)		$p > 0.9999$, ns
						LAP + repeated 0.25% RPV vs LAP + repeated 0.5% RPV (10d)		$p > 0.9999$, ns
7b	% Damaged Area 5d	LAP + repeated saline = 5, LAP + repeated 0.25% RPV = 5, LAP + repeated 0.5% RPV = 5	One-way ANOVA	Treatment: $F (2, 12) = 5.885$, $p = 0.0166$	Tukey's multiple comparison	LAP + repeated saline vs LAP + repeated 0.25% RPV	95%[CI], -16.29 to 14.37	$p = 0.9848$, ns
						LAP + repeated saline vs LAP + repeated 0.5% RPV	95%[CI], -32.86 to -2.201	$p = 0.0253$
						LAP + repeated 0.25% RPV vs LAP + repeated 0.5% RPV	95%[CI], -31.90 to -1.243	$p = 0.0341$
7c	% Damaged Area 10d	LAP + repeated saline = 5, LAP + repeated 0.25% RPV = 5, LAP + repeated 0.5% RPV = 5	One-way ANOVA	Treatment: $F (2, 12) = 6.042$, $p = 0.0153$	Tukey's multiple comparison	LAP + repeated saline vs LAP + repeated 0.25% RPV	95%[CI], -11.27 to 15.09	$p = 0.9216$, ns
						LAP + repeated saline vs LAP + repeated 0.5% RPV	95%[CI], -27.01 to -0.646	$p = 0.0397$
						LAP + repeated 0.25% RPV vs LAP + repeated 0.5% RPV	95%[CI], -28.92 to -2.555	$p = 0.0199$
8b	Number of $CD68^+$/$DAPI^+$ cells/image 5d	LAP + repeated saline = 5, LAP + repeated 0.25% RPV = 5, LAP + repeated 0.5% RPV = 5	One-way ANOVA	Treatment: $F (2, 12) = 23.51$, $p < 0.0001$	Tukey's multiple comparison	LAP + repeated saline vs LAP + repeated 0.25% RPV	95%[CI], -14.94 to 19.08	$p = 0.9438$, ns
						LAP + repeated saline vs LAP + repeated 0.5% RPV	95%[CI], -53.79 to -19.77	$p = 0.0002$
						LAP + repeated 0.25% RPV vs LAP + repeated 0.5% RPV	95%[CI], -55.86 to -21.84	$p = 0.0001$
8c	Number of $MyoD^+$/$DAPI^+$ cells/image 5d	LAP + repeated saline = 5, LAP + repeated 0.25% RPV = 5, LAP + repeated 0.5% RPV = 5	One-way ANOVA	Treatment: $F (2, 12) = 18.18$, $p = 0.0002$	Tukey's multiple comparison	LAP + repeated saline vs LAP + repeated 0.25% RPV	95%[CI], -33.26 to 20.18	$p = 0.7942$, ns
						LAP + repeated saline vs LAP + repeated 0.5% RPV	95%[CI], -81.97 to -28.54	$p = 0.0004$
						LAP + repeated 0.25% RPV vs LAP + repeated 0.5% RPV	95%[CI], -75.43 to -22.00	$p = 0.0010$
